# The effects of omega‐3, DHA, EPA, Souvenaid® in Alzheimer's disease: A systematic review and meta‐analysis

**DOI:** 10.1002/npr2.12455

**Published:** 2024-06-25

**Authors:** Ernesto Calderon Martinez, Stephin Zachariah Saji, Jonathan Victor Salazar Ore, Omar A. Borges‐Sosa, Samyuktha Srinivas, Naga Sai Rasagna Mareddy, Tanseem Manzoor, Mariela Di Vanna, Yasemin Al Shanableh, Rishabh Taneja, Victor Sebastian Arruarana

**Affiliations:** ^1^ Digital Health Universidad Nacional Autónoma de México Mexico City Mexico; ^2^ Our Lady of Fatima University Valenzuela City Philippines; ^3^ Facultad de Ciencias Médicas Universidad de Buenos Aires Buenos Aires Argentina; ^4^ Indiana University Bloomington Indiana USA; ^5^ Manipal Academy of Higher Education Kasturba Medical College Mangalore Karnataka India; ^6^ Department of Radiology University of Alabama at Birmingham Birmingham Alabama USA; ^7^ College of Medicine University of Sharjah Sharjah United Arab Emirates; ^8^ Department of Internal Medicine RWJBH Rutgers Health CMC Toms River New Jersey USA; ^9^ Department of Medical Education Hamad Medical Corporation Doha Qatar; ^10^ Government Medical College and Hospital Chandigarh India; ^11^ Internal Medicine Brookdale University Hospital and Medical Center Brooklyn New York USA

**Keywords:** Alzheimer's disease, cognitive decline, DHA, Omega‐3 fatty acids, Souvenaid®

## Abstract

**Background:**

Alzheimer's disease (AD) is the most common cause of dementia worldwide. Omega‐3 fatty acids (n‐3‐PUFA) are essential to normal neural development and function. Souvenaid®, a medical supplement that contains n‐3‐PUFA's: eicosatetraenoic acid (EPA) and docosahexaenoic acid (DHA), has emerged as an alternative, slowing cognitive decline in AD patients. In this study, we investigated the effect of dietary supplementation with n‐3‐PUFA, EPA, DHA, and Souvenaid® in AD patients.

**Aim:**

This systematic review and meta‐analysis aim to establish the relationship between n‐3‐PUFA, EPA, DHA, and Souvenaid® with cognitive effects, ventricular volume and adverse events in AD patients.

**Methods:**

A systematic search of randomized control trials (RCT), cohorts, and case–control studies was done in PubMed, Scopus, Web of Science, Cochrane, and Embase for AD adult patients with dietary supplementation with n‐3‐PUFA, EPA, DHA, or Souvenaid® between 2003 and 2024.

**Results:**

We identified 14 studies with 2766 subjects aligned with our criteria. Most publications described positive cognitive outcomes from supplements (58%). The most common adverse events reported were gastrointestinal symptoms. CDR scale showed reduced progression of cognitive decline (SMD = −0.4127, 95% CI: [−0.5926; −0.2327]), without subgroup differences between different dietary supplement interventions. ADCS‐ADL, MMSE, ADAS‐cog, adverse events, and ventricular volume did not demonstrate significant differences. However, Souvenaid® showed a significant negative effect (SMD = −0.3593, 95% CI: −0.5834 to −0.1352) in ventricular volumes.

**Conclusions:**

The CDR scale showed reduced progression of cognitive decline among patients with n‐3‐PUFA supplemental interventions, with no differences between different n‐3‐PUFA supplements.

## Abstract 2

Supplementation with n‐3 PUFA shows potential in slowing cognitive decline in AD patients, as indicated by the CDR scale. However, our findings suggest no clear distinctions between various n‐3 PUFA supplements. These results highlight both the potential benefits and limitations of dietary interventions in managing cognitive decline in AD patients.

## Graphical abstract

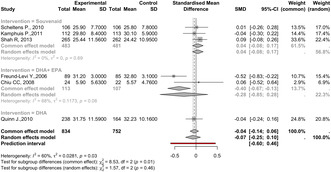

## 1 INTRODUCTION

Alzheimer's disease (AD) is a multifactorial, progressive and irreversible neurodegenerative disorder. The biological markers of β‐amyloid and tau neurofibrillary tangles are defining features of this disease.^1^, ^2^ Unfortunately, AD remains a leading cause of dementia, affecting 27 million people worldwide (60–70% of all dementia cases)^3^ and over 6 million Americans in 20 234 numbers are expected to grow 152 million and 27 million by 2050, respectively. The global cost of dementia‐related care is $1 trillion annually, and no known cure currently exists to modify the course of AD.^4^, ^5^

Some risk factors for AD include age, genetics, family history, diabetes, hypertension, obesity, and dyslipidemia.^1^, ^3^, ^6^ The prevalence of AD is correlated with age: 5.0% aged 65–74, 13.1% of those 75–84, and 33.3% of those 85 years of age and above.^7^ Disturbances in Omega‐3 fatty acids (n‐3 PUFA) which include alpha‐linolenic acid (ALA), eicosapentaenoic acid (EPA), docosahexaenoic acid (DHA) levels, lipid rafts, and phospholipid composition are observed in AD.^8^

N‐3 PUFA are essential nutrients obtained from the diet, usually found in fatty fish and fish oil supplements. They are essential to the retina and brain, and myocardium cellular membranes.^9^, ^10^ DHA, a n‐3 PUFA, has been shown to be essential in normal neuronal development particularly retina and neuronal cellular membrane by changing the physical properties of membranes.^10^, ^11^ The brain contains large amounts of n‐3 PUFA, predominantly DHA, which has a half‐life of 2.5 years in the brain, suggesting functional brain changes with n‐3 PUFA deprivation.^12^ Meanwhile, EPA has significant anti‐inflammatory effects protective of the cellular membrane. It directly inhibits proinflammatory markers including IL‐1B and IL‐6.^13^, ^14^

There are limited options for AD patients' cognitive decline some dietary supplements have emerged as a possible treatment measure.^8^ Souvenaid®, a medical supplement intended for AD patients, which includes several vitamins and n‐3 PUFA has shown a slowed decline in cognition, brain atrophy, and disease progression in patients with AD. This systematic review and meta‐analysis aim to investigate the efficacy of n‐3 PUFA along with Souvenaid®, in managing AD and explore their impact on cognition, ventricular volume and adverse effects.^8^, ^12^

## 2 METHODS

The present study employed the Preferred Reporting Items for Systematic Review and Meta‐Analysis (PRISMA) 2020 guidelines to conduct a comprehensive systematic review.^15^, ^16^

### 2.1 Searching methods

Our search encompassed PubMed, Scopus, Web of Science, Cochrane, and Embase using Medical Subject Headings (MeSH) terms and free text terms on January 29, 2024 (see Data S1). We adhered to a PRISMA flowchart^15^ to guide the systematic review article selection process, resulting in a uniform dataset and enhancing the accuracy and reliability of our findings.

## 3 CRITERIA FOR CONSIDERING STUDIES IN THIS REVIEW

### 3.1 Types of study

For our research study, the effects of omega‐3, DHA, EPA, Souvenaid® in Alzheimer's disease, we systematically reviewed relevant studies published from 2003 to 2024. The selected years capture significant advancements and emerging research trends in omega‐3 fatty acid supplementation and Alzheimer's disease. Available in English and Spanish. This systematic review included studies that met the following inclusion criteria: RCT, cohort, and case–control studies reporting the effects of omega‐3 fatty acid, DHA, EPA, and Souvenaid® (medical, nutritional drink with DHA, EPA, and more nutrients) in Alzheimer's disease. We excluded case reports, case series, dissertations, book chapters, protocol articles, reviews, news articles, conference abstracts, letters to the editor, editorials, and comment publications. Furthermore, we excluded studies that did not clearly describe their operationalization, duplicates, and those for which we could not obtain the necessary data or receive a response from the original author via email.

### 3.2 Types of participants

This study has set specific participant selection criteria, including both genders. The focus will be on adults who have Alzheimer's disease. Including only articles that report the effects of omega‐3 fatty acid, DHA, EPA, and Souvenaid® (medical, nutritional drink with DHA, EPA, and more nutrients); exclude studies involving pediatric populations (under 18 years of age). The study aims to include a variety of participants to gain a better understanding of the intervention.

### 3.3 Types of intervention

To be eligible for inclusion in this study, the selected research must evaluate the effect of Omega‐3, DHA, EPA, and nutritional supplement Souvenaid® in Alzheimer's disease adult patients. The interventions may include oral supplements or any other consumption way. The control group can receive no intervention, standard care, or alternative intervention. Exclude studies that do not involve the administration of Omega‐3, DHA, EPA, and nutritional supplement Souvenaid® in any subgroups or groups.

### 3.4 Outcomes

The outcomes to be measured included studies that report relevant outcomes, specifically effect on cognition, assessed by Alzheimer's Disease Cooperative Study – Activities of Daily Living Scale (ADCS‐ADL), Mini‐Mental Scale Examination (MMSE), Alzheimer's Disease Assessment Scale‐cognitive (ADAS‐cog) and Clinical Dementia Rating (CDR) Scale; Ventricular Volume assessed by MRI; Adverse Effects and exclude studies that do not report information related to cognitive impairment.

### 3.5 Selection of studies

After an initial screening of titles and abstracts, two reviewers (JVSO, NRSM) independently chose trials for inclusion in this review based on predetermined criteria. The search was conducted using Rayyan,^17^ with relevant data extracted and duplicates filtered. Keywords were utilized to identify inclusion and exclusion criteria‐related terms on Rayyan (see Data S1). Any disagreements regarding study inclusion were resolved through consensus and consultation with a third reviewer (ECM).

Following this, a full‐text analysis was undertaken, with two reviewers (JVSO, OABS) independently selecting trials for inclusion based on the predetermined criteria. Any disagreements on study inclusion were settled through consensus and consultation with a third reviewer (SZS).

### 3.6 Data evaluation

We conducted data evaluation according to the criteria outlined by Cochrane. We used the Cochrane RoB 2.0 tool for randomized controlled trials (RCTs)^17^ and the Newcastle Ottawa Scale for Cohort and case–control studies to assess study quality in the systematic review.^18^ Two independent reviewers assessed bias risk in each study (JVSO, SZS), adhering to the specific criteria and guidelines of the respective tools. Any reviewer disagreements were resolved through discussion with a third, blinded reviewer (ECM).

The methodological aspects of trials and case–control studies were categorized as having low, high, or unclear risk of bias following the Cochrane Handbook for Systematic Reviews of Interventions^19^ and NOS guidelines,^20^ respectively. Details regarding any downgrading or upgrading of evidence quality will be presented in the summary of findings table, providing transparency and explanations for bias assessment in each study included.

### 3.7 Statistical analysis

Meta‐analysis was performed using the R Software version 023.09.1 + 494 (2023.09.1 + 494) to calculate the effect size.^21^ Effect sizes were presented as mean differences with 95% confidence intervals (CI). The random‐effects model was used for pooling analysis to compensate for the heterogeneity of studies^22^, ^23^ statistics. In this regard, *I* ^2^ ≥ 50% and ≥75% indicated substantial heterogeneity^23^ study removal method to the sub‐analysis to assess whether any individual study exerted particular influence on the overall effect size,^24^, ^25^ *p*‐values <0.05 were considered statistically significant.

## 4 RESULTS

**FIGURE 1:**
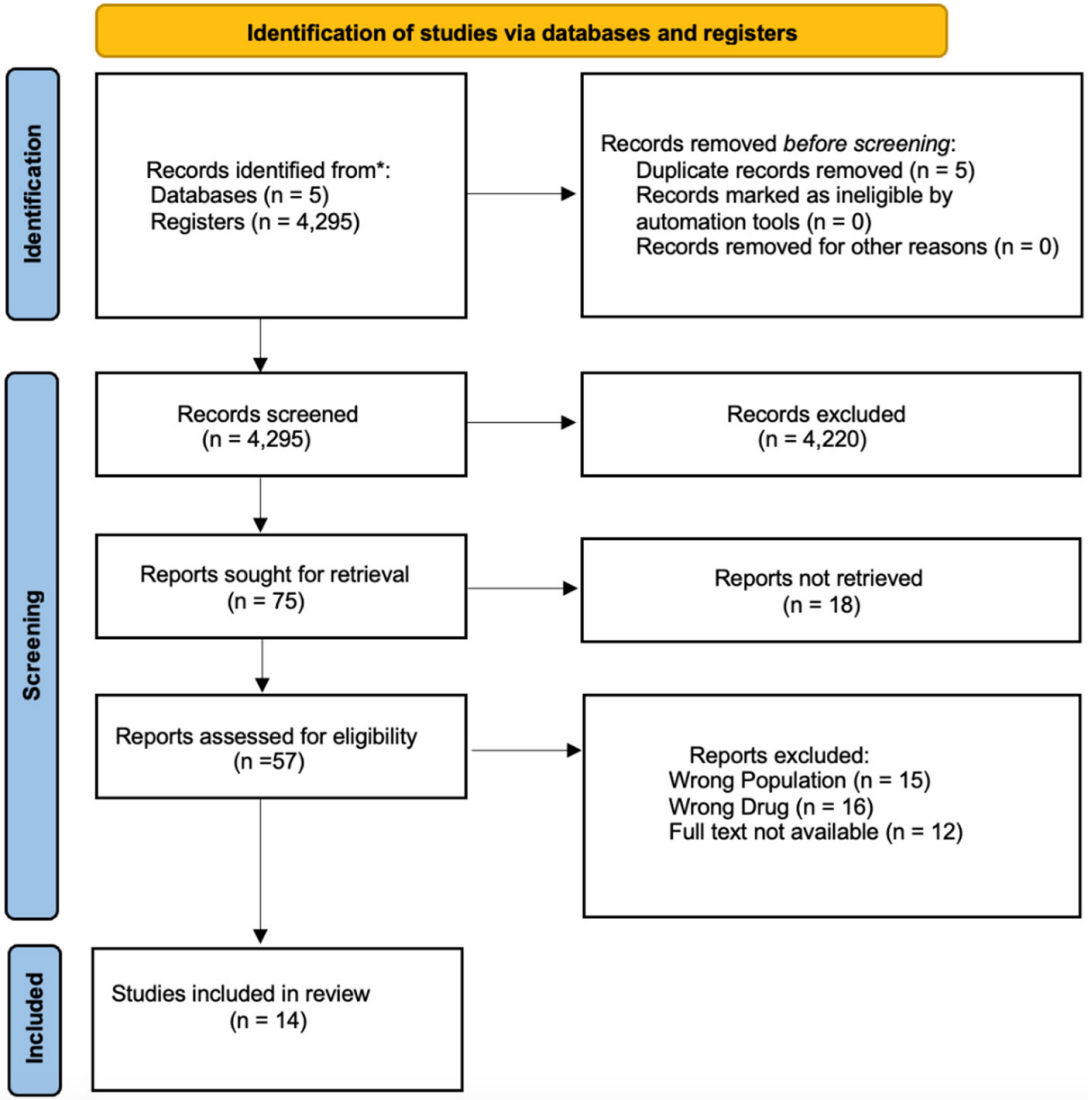
PRISMA flow diagram.

Across the database, we identified 4295 possible articles using five total databases. After a thorough examination, five duplicate articles were removed before screening. During the screening, 75 publications were sought for retrieval, and 18 were further removed in the screening process. Out of the remaining, 57 publications were assessed for screening eligibility, and 14 were assessed and included in the final review process. The total sample size of the 14 publications was 2766 participants (Figure 1).

**FIGURE 2:**
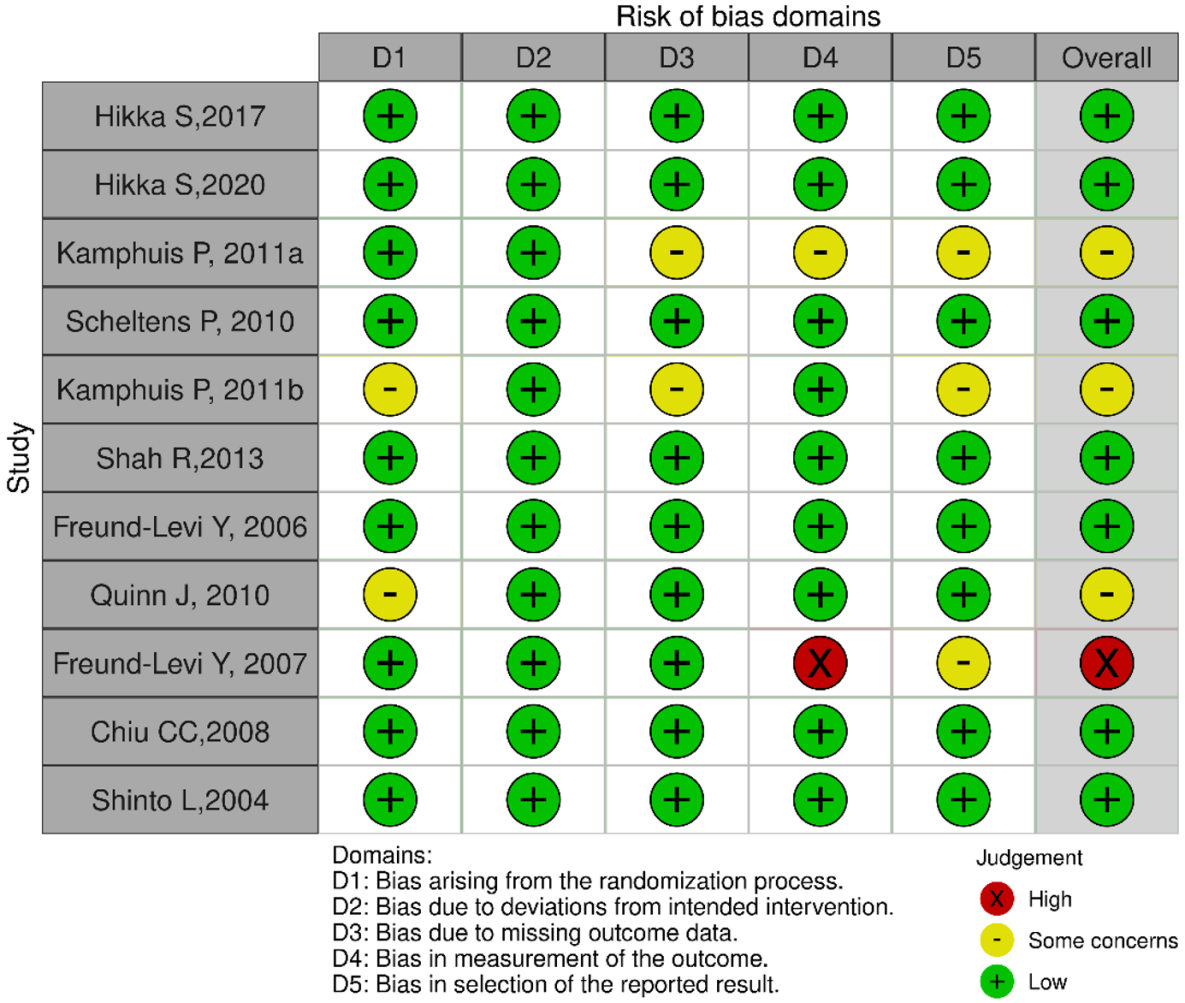
Risk of bias traffic light plot.

This risk of bias assessment used Cochrane's Risk of Bias 2.0 tool for randomized control trials to assess the quality or risk of bias of the 11 included studies. Risk of bias traffic light plot and bar plot were created using the tool ROBVIS.^26^ Our results summarized in Figure 2 show that one article (9%) showed a high risk of bias, while three (28%) showed some concerns, and the remaining seven (63%) showed a low risk of bias. Our selection showed that most of our publications resulted in low risk to some concern, with only one article (9%) in the red high‐risk label. The remainder of the publications, both prospective and retrospective studies, used the New Castle‐Ottawa Scale (NOS). Our selection showed that three articles (100%) of the studies were of Good Quality.

The primary outcome obtained from the selected research papers focuses on the effect of Omega‐3, DHA, EPA, and nutritional supplement Souvenaid® interventions in older patients with Alzheimer's disease, emphasizing the effect on cognition measured by different cognitive function measures (MMSE, ADAS‐Cog, CDR, ADCS‐ADL). These participants and studies were from a wide geographic range, including diverse countries such as Finland, the Netherlands, Japan, Sweden, the USA, the United Kingdom, and Taiwan.

While the selected publications had varied results, most saw a positive effect on cognition, using Omega 3 interventions compared to placebo. Of the selected publications (58%) saw a positive effect on cognition using Omega 3 interventions, while the remaining (42%) saw no significant difference. This information is summarized in Table 1.

**TABLE 1:** General outcomes of included studies.

Author	Year	Study design	Age	Sample size (total)	Follow‐up period	Result
Hikka S.^28^	2017	RCT	N/A	311	24 months	The multinutrient intervention had no significant effect on the Neuropsychological Test Battery primary endpoint over 2 years in prodromal Alzheimer's disease, although potential benefits were seen on the cognitive‐functional measure CDR‐SB and brain atrophy measures. We observed significantly less increase in ventricular volume (*p* = 0.046)
Hikka S.^29^	2020	RCT	N/A	81	36 months	This multinutrient intervention slowed decline on clinical and other measures related to cognition, function, brain atrophy, and disease progression. These results indicate that intervention benefits increased with long‐term use
Kamphuis P.^33^	2011	RCT	N/A	225	6 months	Patients with lower BMI at baseline may benefit more from Souvenaid®, with respect to functional outcome, than those with higher baseline BMI. Baseline BMI is a predictor of ADL outcome
Yamazaki T.^31^	2015	Prospective	78.6 ± 7.2	133	12 months	Long‐chain omega‐3 PUFA is closely associated with cognitive function, and the EPA/AA ratio can be regarded as a predictive marker for the preservation of cognitive ability in elderly AD patients
Scheltens P.^34^	2010	RCT	73.7 ± 7.51	212	3 months	This proof‐of‐concept study showed that supplementation with the multinutrient drink Souvenaid® for 12 weeks is well‐tolerated and results in an improvement in memory in patients with mild AD
Kamphuis P.^30^	2011	RCT	N/A	225	6 months	Overall, intake adherence was significantly correlated with ADAS‐cog improvement in the active product group (correlation coefficient = −0.260; *p* = 0.019), but not the control group
Eriksdotter M.^35^	2015	Retrospective Cohort	74 ± 9	165	6 months	Preservation of cognitive functioning, assessed by ADAS‐cog or its sub‐items (but not MMSE) scores, was significantly associated to increasing plasma omega 3 FA levels over time
Shah R^36^	2013	RCT	76.7 ± 8.2	527	6 months	Cognitive performance as assessed by ADAS‐cog showed decline over time in both control and active study groups, with no significant difference between study groups
Freund‐Levi Y.^27^	2006	RCT	72.5 ± 8.9	174	12 months	At 6 months, the decline in cognitive functions did not differ between the groups. However, in a subgroup (*n* = 32) with very mild cognitive dysfunction (MMSE >27 points), a significant (*p* < 0.05) reduction in MMSE decline rate was observed in the ω‐3 fatty acid‐treated group compared with the placebo group
Quinn J.^37^	2010	RCT	76 ± 8.7	402	18 months	Supplementation with DHA compared with placebo did not slow the rate of cognitive and functional decline in patients with mild to moderate Alzheimer disease
Freund‐Levi Y.^38^	2007	RCT	N/A	204	12 months	Supplementation with omega3 in patients with mild to moderate AD did not result in marked effects on neuropsychiatric symptoms except for possible positive effects on depressive symptoms
Boston P.^32^	2004	Prospective	N/A	22	6 months	No statistic/clinical difference in treatment effect of ethyl‐EPA on cognition during this 12‐week study
Chiu CC^39^	2008	RCT	N/A	46	6 months	Omega 3 PUFA can improve general clinical function but not cognitive function
Shinto L.^40^	2014	RCT	N/A	39	12 months	In a small pilot study combining ω‐3 with LA slowed both cognitive and functional decline in mild to moderately impaired AD participants over 12 months, and the combination appears to be safe at the doses evaluated

The review highlighted varied outcomes in cognitive and functional measures. Two Studies employing the Clinical Dementia Rating scale yielded conflicting results^27^, ^28^; one found no significant differences at 12 months,^27^ while the other reported less worsening in the treatment group at 24 months.^28^

The efficacy of Omega‐3 fatty acids (EPA and DHA) was mixed. Two studies found no significant improvement in MMSE scores, suggesting a limited impact on cognitive function.^28^, ^29^ However, another study observed benefits in mild Alzheimer's cases, suggesting potential cognitive improvements in the early stages, implying potential early‐stage cognitive improvements.^30^

For ADAS‐cog scores, the results were inconsistent. One study reported improvements with Souvenaid® in patients with higher baseline scores,^31^ while another found no significant effects of EPA and DHA.^28^ Overall, these studies present a nuanced picture of Alzheimer's interventions, with some showing benefits in specific cognitive aspects while others indicate no significant change.

Adverse events were reported in 9 out of 14 publications included in our paper. Of those, two publications did not report adverse events compared to a control or placebo group.^27^, ^32^ In the remaining seven publications, two are identified as continuing the same research study^28^, ^29^ six studies were included in the review of reported adverse events. All six of the research studies have reported no statistically significant difference in the incidence of adverse events between active and control groups. In the nine publications that reported adverse events, the most reported adverse events involved the gastrointestinal.

## 5 META‐ANALYSIS RESULT

### 5.1 Adverse events

This meta‐analysis assessed adverse events from Omega‐3 fatty acid supplements in six studies (1184 observations). The relative risk (RR) was 1.0149 (95% CI: 0.9624–1.0702, *p* = 0.5861), indicating no significant risk increase (see Data S1). Low heterogeneity was confirmed by a tau‐squared value (<0.0001) and an *I*‐squared value of 17.4% (95% CI: 0.0–62.2%).

#### 5.1.1 Subgroup and sensitivity analysis

Different Omega‐3 forms showed variable RRs, but none indicated a significant risk increase. Moderate residual heterogeneity (*I* ^2^ = 55.45%) was observed. Sensitivity analysis was not performed due to the low heterogeneity.

#### 5.1.2 Publication bias

A linear regression test was not conducted due to the limited number of studies. Funnel plot symmetry suggested no publication bias (see Data S1).

### 5.2 Cognitive decline rating (CDR) scale

**FIGURE 3:**
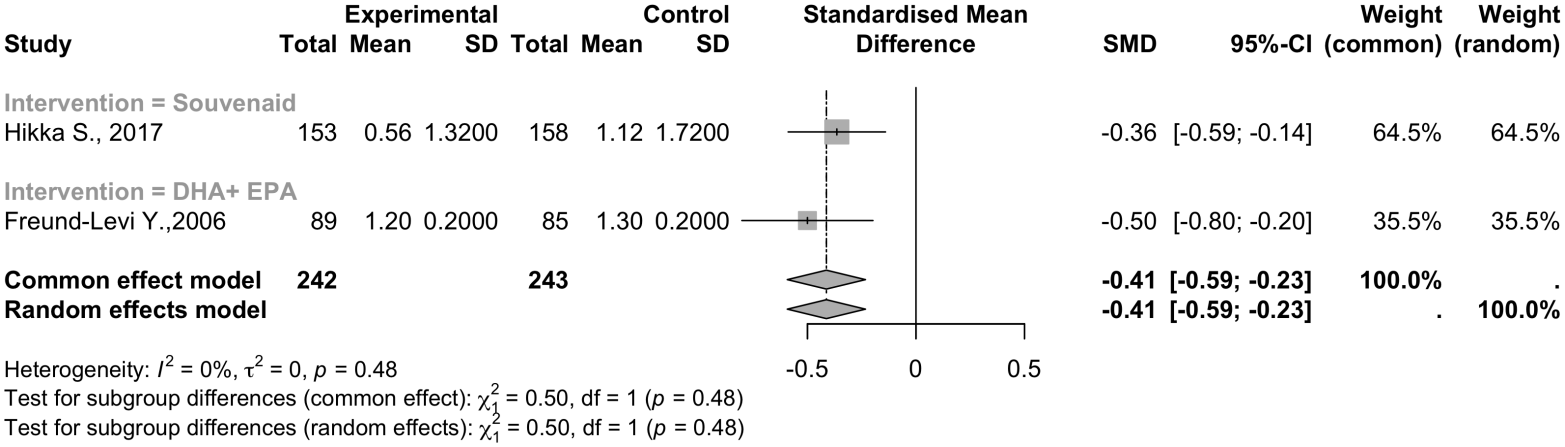
Forest plot detailing mean difference and 95% confidence intervals (CI) for the effect of different Omega 3 types against Placebo on the CDR scale. Forest plot illustrating the Standardized Mean Difference (SMD) on the CDR scale between two studies under the random effect model, indicating Omega‐3 supplements significantly reduced the progression of cognitive decline with minimal heterogeneity among studies (*I* ^2^ = 0%).

Two studies involving 485 participants showed that Omega‐3 supplements significantly reduced the progression of cognitive decline (SMD = −0.4127, 95% CI: [−0.5926; −0.2327]). Heterogeneity among studies was minimal, with an *I* ^2^ value of 0.0% and tau^2^ of 0 (See Figure 3).

#### 5.2.1 Subgroup and sensitivity analysis

Different Omega‐3 types, such as Souvenaid® and DHA + EPA, demonstrated similar benefits. No significant subgroup differences were found (*Q* = 0.50, df = 1, *p* = 0.4801). Sensitivity analysis was not conducted due to the lack of heterogeneity.

#### 5.2.2 Publication bias

The number of studies precluded a linear regression test, but funnel plot symmetry indicated no publication bias (see Data S1).

### 5.3 ADCS‐ADL

**FIGURE 4:**
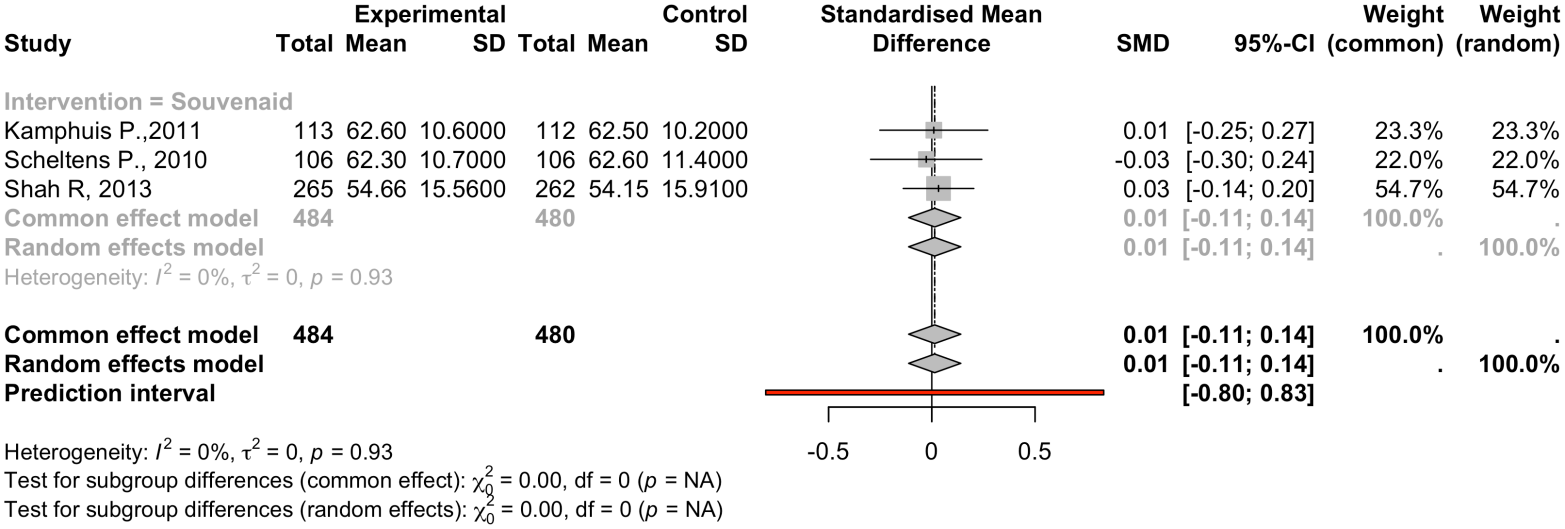
Forest plot detailing mean difference and 95% confidence intervals (CI) for the effect of different Omega 3 types against Placebo on ADCS‐ADL scores. Forest plot illustrating the Standardized Mean Difference (SMD) on ADCS‐ADL scores across three studies under random effect model, indicating Omega‐3 supplements had minimal nonstatistical important effect on ADCS‐ADL scores with minimal heterogeneity among studies (*I* ^2^ = 0%).

In three studies totaling 964 observations, omega‐3 supplements had minimal nonstatistical important effects on ADCS‐ADL scores (SMD = 0.0140, 95% CI: −0.1123 to 0.1403). Heterogeneity was negligible, with tau^2^ of 0 and *I* ^2^ of 0.0% (95% CI: 0.0–89.6%; See Figure 4).

#### 5.3.1 Subgroup and sensitivity analysis

Due to insufficient data, no subgroup analysis was conducted. Sensitivity analysis was not performed, given the low heterogeneity.

#### 5.3.2 Publication bias

The limited number of studies made a linear regression test unfeasible. Nevertheless, funnel plot symmetry suggests no publication bias (see Data S1).

### 5.4 Ventricular volume

**FIGURE 5:**
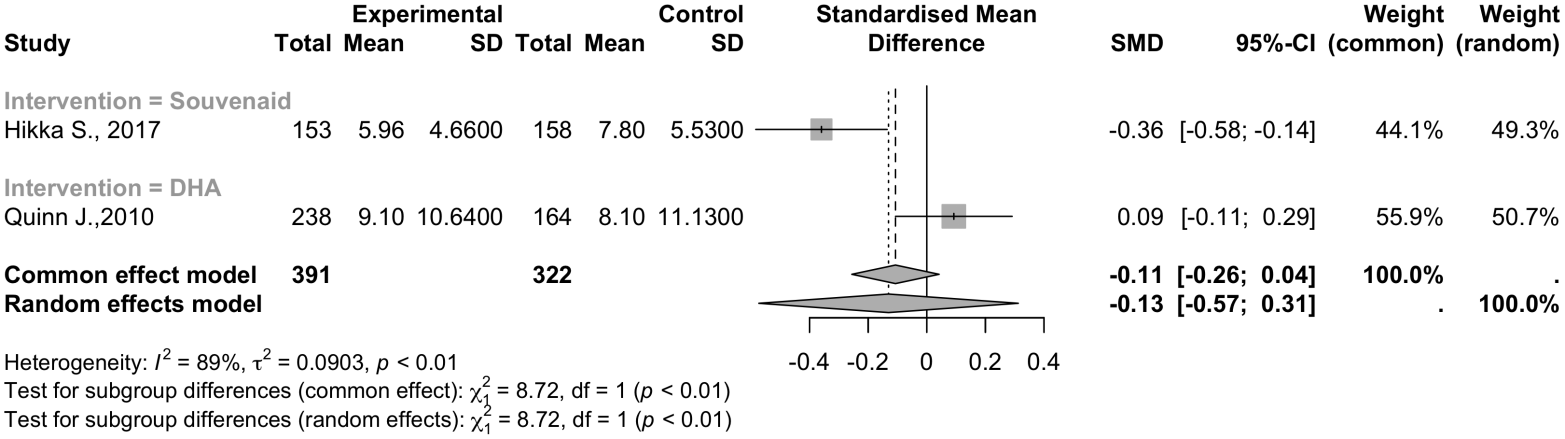
Forest plot detailing mean difference and 95% confidence intervals (CI) for the effect on ventricular volume by different Omega 3 types against Placebo. Forest plot illustrating the Standardized Mean Difference (SMD) of Ventricular volume between two studies under random effect model, SMD for ventricular volume changes was −0.1305 (95% CI: −0.5730 to 0.3120, *p* = 0.5633) with high heterogeneity among studies (*I* ^2^ = 88.5%).

This meta‐analysis included two studies with 713 observations (391 in experimental groups and 322 in control groups). The standardized mean difference (SMD) for ventricular volume changes was −0.1305 (95% CI: −0.5730 to 0.3120, *p* = 0.5633). The heterogeneity among studies was high, with tau^2^ at 0.0903 and an *I* ^2^ value of 88.5% (95% CI: 56.4–97.0%; See Figure 5).

#### 5.4.1 Subgroup and sensitivity analysis

Subgroup analyses compared different interventions (Souvenaid® vs. DHA). Souvenaid® showed a significant negative effect (SMD = −0.3593, 95% CI: −0.5834 to −0.1352), whereas DHA showed a nonsignificant positive effect (SMD = 0.0922, 95% CI: −0.1068 to 0.2912). The test for subgroup differences was significant (*Q* = 8.72, df = 1, *p* = 0.0031). Sensitivity analysis was not conducted due to the limited number of studies.

#### 5.4.2 Publication bias

The linear regression test was impossible due to the small number of studies. However, funnel plot asymmetry suggests potential publication bias (see Data S1).

### 5.5 Alzheimer's disease assessment scale (ADAS)

**FIGURE 6:**
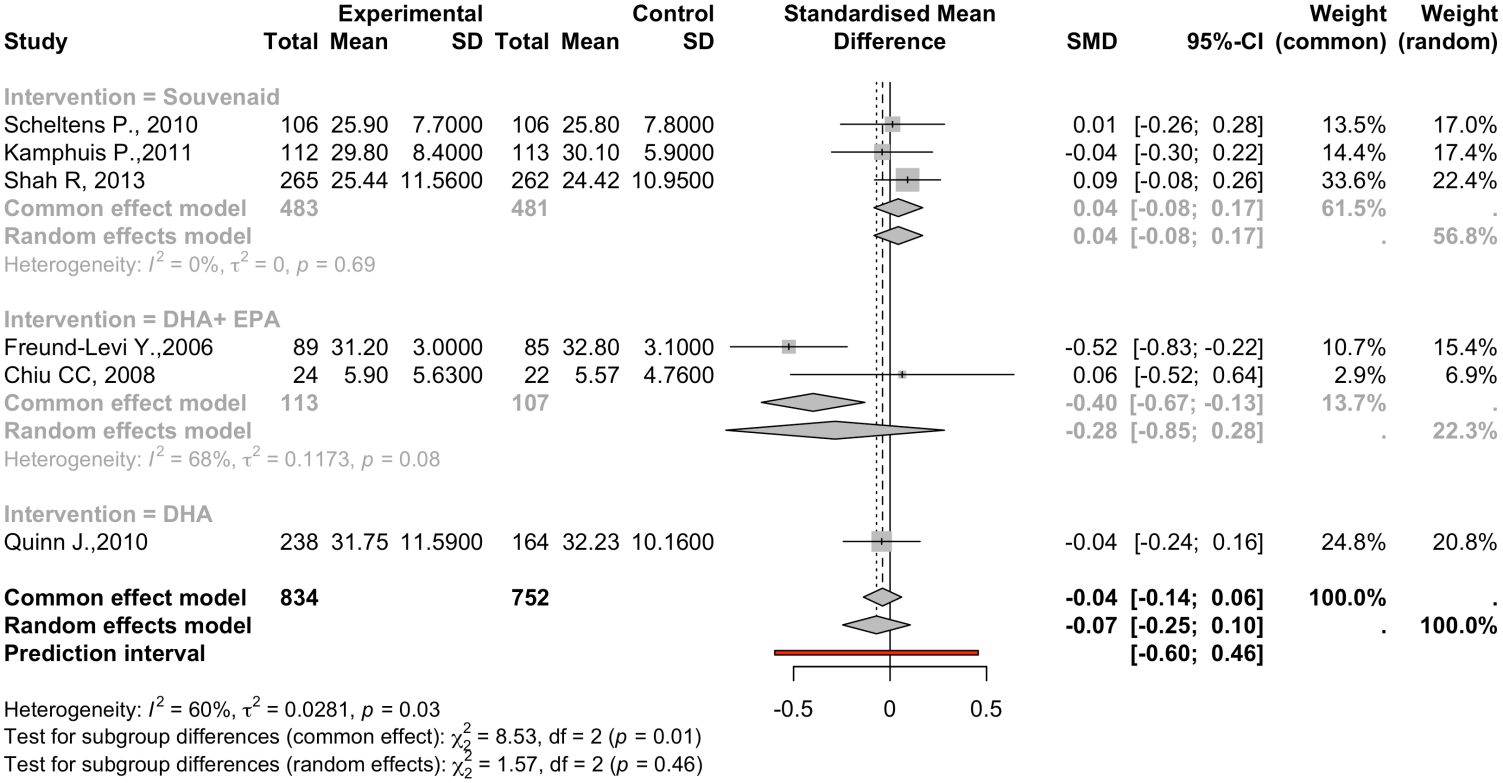
Forest plot detailing mean difference and 95% confidence intervals (CI) for the effect of different Omega 3 types against Placebo on ADAS scores. Forest plot illustrating the Standardized Mean Difference (SMD) on ADAS scores across six studies under the random effect model, SMD of −0.0702 (95% CI: −0.2454 to 0.1049, *p* = 0.4320) with moderate to high heterogeneity among studies (*I* ^2^ = 59.6%).

Involving six studies with 1586 observations (834 in experimental and 752 in control groups), the analysis showed an SMD of −0.0702 (95% CI: −0.2454 to 0.1049, *p* = 0.4320). The heterogeneity was moderate to high, with tau^2^ at 0.0281 (95% CI: 0.0000–0.2855) and an *I* ^2^ of 59.6% (95% CI: 0.8–83.6%; See Figure 6).

#### 5.5.1 Subgroup and sensitivity analysis

Subgroup analyses evaluated different interventions (Souvenaid®, DHA, DHA + EPA). DHA + EPA showed a nonsignificantly negative effect (SMD = −0.2847, 95% CI: −0.8510 to 0.2815), whereas Souvenaid® and DHA alone showed no significant effects. The subgroup differences test was insignificant (*Q* = 1.57, df = 2, *p* = 0.4552). Sensitivity analysis was not performed due to the complex nature of interventions and heterogeneity levels.

#### 5.5.2 Publication bias

The linear regression test was not conducted due to the limited number of studies, and the funnel plot asymmetry indicated possible publication bias (see Data S1).

### 5.6 Mini‐mental state examination (MMSE)

**FIGURE 7:**
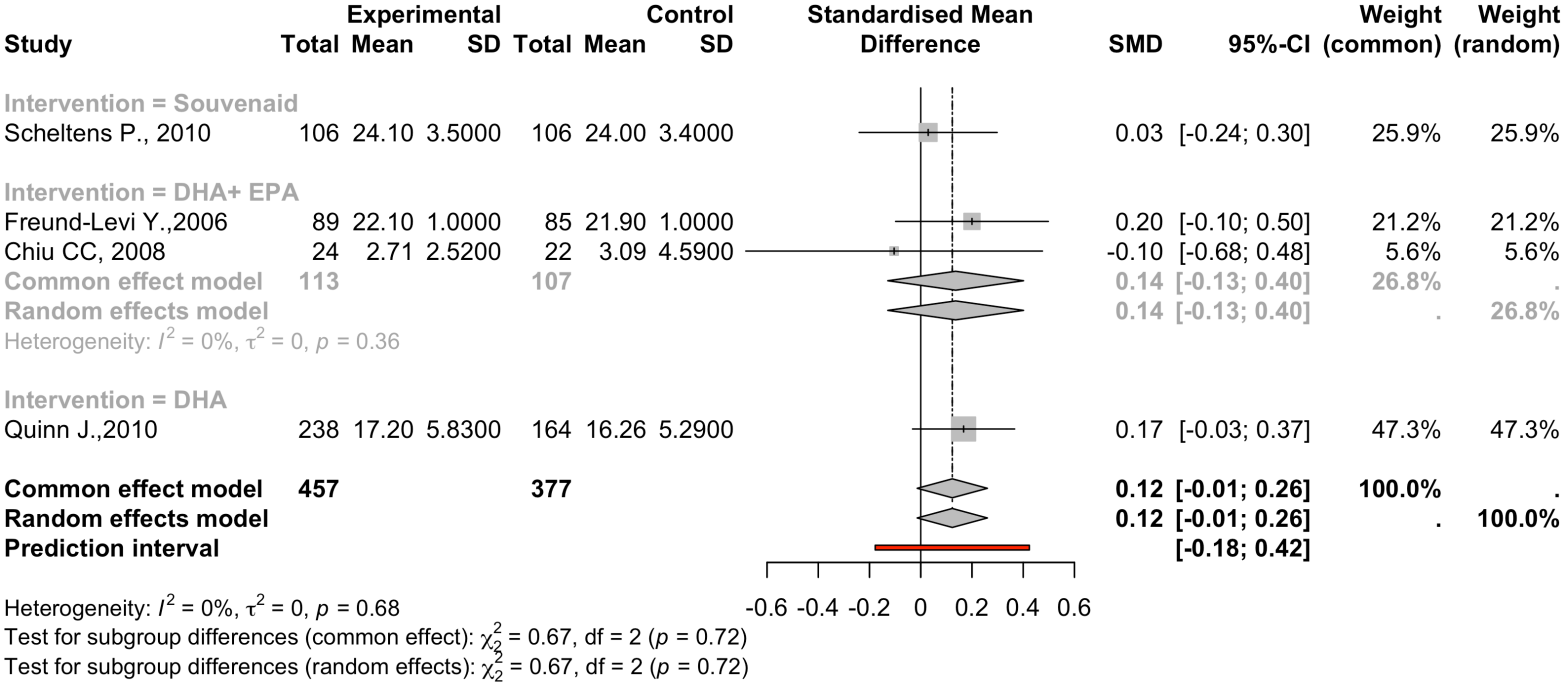
Forest plot detailing mean difference and 95% confidence intervals (CI) for the effect of different Omega 3 types against Placebo on MMSE scores. Forest plot illustrating the Standardized Mean Difference (SMD) on MMSE scores across four studies under the random effect model, SMD of 0.1232 (95% CI: −0.0139 to 0.2603, *p* = 0.0781) with minimal heterogeneity among studies (*I* ^2^ = 0%).

This meta‐analysis incorporated data from four studies with 834 observations (457 in experimental groups and 377 in control groups). The analysis yielded an SMD of 0.1232 (95% CI: −0.0139 to 0.2603, *p* = 0.0781). Heterogeneity was minimal, with tau^2^ at 0 and an *I* ^2^ of 0.0% (95% CI: 0.0–84.7%; See Figure 7).

#### 5.6.1 Subgroup and sensitivity analysis

Different interventions (Souvenaid®, DHA, DHA + EPA) were considered. The DHA group showed a small, nonsignificant positive effect (SMD = 0.1674, 95% CI: −0.0319 to 0.3666). No significant effects were observed for Souvenaid® and DHA + EPA. The subgroup differences test was insignificant (*Q* = 0.67, df = 2, *p* = 0.7158). Sensitivity analysis was not conducted due to the consistent effects and low heterogeneity.

#### 5.6.2 Publication bias

A linear regression test was not feasible because of the limited number of studies. The funnel plot indicated no significant publication bias (see Data S1).

## 6 DISCUSSION

Alzheimer's is an irreversible, progressive neurodegenerative disorder and the primary cause of dementia, affecting approximately 27 million people worldwide.^1^, ^2^ The lack of discovered treatment to halt the progression of functional and cognitive decline continues to compound the burden of society. This systematic review and meta‐analysis, which analyses data from 14 articles, aims to establish the role of dietary supplements such as n‐3 PUFA (DHA and EPA) and Souvenaid® in improving cognition, quality of life, and other parameters in patients with AD.^8^, ^12^ We have used certain cognitive parameters such as the CDR scale, ADCS‐ADL score, ADAS‐cog score, and MMSE to assess the impact of the intervention on cognition, while MRI assessed ventricular volume. Most selected publications (58%) reported a positive effect on cognition with omega‐3 interventions, while the remaining (42%) observed no significant difference. This indicates that while omega‐3 interventions might benefit specific individuals, they may not offer benefits across the board. Considering individual patient characteristics and preferences is essential when deciding on a treatment plan.

Nine articles reported adverse effects from Omega‐3 fatty acid supplementation. None of these studies posed a statistically significant difference between the active and control groups. We excluded three articles for quantitative analysis due to insufficient data. The most common adverse events were gastrointestinal symptoms; this was not analyzed in the meta‐analysis as it was not one of the main outcomes of interest.

The review of Ventricular volumes in the brain stated a positive effect of intervention with significantly less volume reduction and a lower rate of deterioration among the active group of patients.^26^ However, the meta‐analysis found it statistically insignificant, with an estimated mean difference of −0.1305 (95% CI: −0.5730 to 0.3120, *p* = 0.5633) with high heterogeneity. Intervention with Souvenaid® showed a significant negative effect on the volumes, whereas DHA showed a nonsignificant positive effect, conveying a difference in outcome among the type of dietary intervention. Subgroup analysis suggests that Souvenaid® and DHA may affect ventricular volume changes differently. The difference observed between interventions underscores the importance of future research in explaining the underlying mechanisms. Clinicians should consider the differences in the intervention and closely monitor patients while receiving interventions with regular imaging assessments and clinical evaluation.

The review of the Clinical Dementia Rating scale observed varied outcomes in cognitive and functional measures. Meta‐analysis of the CDR scale showed that nutritional intervention significantly reduced the progression of cognitive decline in patients with AD (SMD = −0.4127, 95% CI: [−0.5926; −0.2327]). A significant reduction in the progression of cognitive decline suggests that the intervention could serve as an option for individuals at risk of cognitive impairment. However, it is crucial to recognize that various cognitive scales may measure different aspects of cognitive function and can have differing sensitivity to changes in cognitive abilities. Future research is necessary to enhance our understanding of the specific effect of intervention and long‐term implication among various population and cognitive domain.

ADAS‐cog score showed variable response to intervention, with a few articles reporting improved cognition and the others reporting no change.^31^, ^33^, ^34^ The group receiving DHA showed a small yet nonsignificant positive effect on the MMSE, while none were observed with Souvenaid® and EPA. The meta‐analysis found no significant overall effect on ADAS scores across the studies. Subgroup analyses indicated that DHA + EPA had a nonsignificantly negative effect, while Souvenaid® and DHA alone did not exhibit substantial effects. Considering the moderate to high heterogeneity observed, it is advisable to interpret the results cautiously. The clinical importance of these findings emphasizes the need for further research aimed at enhancing our understanding of the effects of different interventions on ADAS scores in individuals with Alzheimer's disease.

Most of the selected articles resulted in a low risk of bias, with only one article (9%) having a high risk of bias. This leads us to believe that our conclusions from the articles are reliable. Asymmetry observed in the funnel plots assessing Ventricular Volumes and ADAS Score signifies a potential Publication Bias in the respective cognition parameters with an overestimated effect size. Our qualitative findings indicate that nutritional supplementation with Omega‐3 fatty acids appeared to decelerate cognitive decline and enhance overall well‐being in patients with Alzheimer's disease (AD). However, our quantitative analysis did not reveal a statistically significant difference between the active and control groups, contradicting these qualitative observations. Healthcare providers should interpret qualitative findings cautiously, acknowledging that they may not always correlate with quantitative analysis. While qualitative data offer valuable insights, quantitative analysis is essential for establishing statistical significance and treatment efficacy. Despite the lack of statistical significance in quantitative analysis, healthcare providers should consider individual patient characteristics, preferences, and responses to intervention.

Scouring through Systematic Reviews and meta‐analyses performed in the previous years on related subjects taught us that most studies addressed populations with mild to moderative cognitive dysfunction in AD rather than severe levels of impairment. A previous Meta‐analysis showed positive effects on cognition in long‐term (minimum period of 10% of total life span) supplementation with omega‐3 FA on mice models with advanced AD.^41^ In addition, this study suggests differential effects according to gender, showing a larger diminished neurodegeneration in female animals. Another previous quantitative study supports a positive relationship between a longer follow‐up duration and a stronger protective effect of higher fish intake against the risk of AD.^42^ Despite these studies suggesting that supplementation with Omega‐3 FA slowed down cognition decline, especially in the long term, they did not find statistically significant evidence of this protective effect on humans.

Additionally, heterogeneity among studies raises concerns about the consistency of these findings. This statement is underpinned by a previous Meta‐analysis, which sustains that there is no consistent evidence to support the effectiveness of Omega‐3 supplementation on cognition in AD in the short and medium term and that supplementation only improves certain aspects of cognitive function in patients with cognitive impairment not associated with dementia.^43^ Our study assesses the impact of multiple nutritional supplements such as Omega‐3 s, DHA, EPA, and Souvenaid® on cognitive parameters. Such a detailed review has not been done in the recent past. Although our Systematic Review and Meta‐analysis found positive effects on Souvenaid® supplementation in decelerating cognitive decline and enhancing overall well‐being in patients with AD, it was not enough to reach a statistical significance between the active and control groups. These results may be due to the lack of a common strategy to report improvement and the few articles we have included for our strict criteria. Nevertheless, healthcare providers should consider these outcomes when preventing and treating AD.

## 7 LIMITATIONS

Our review focused on articles published in English and Spanish, Randomized Control Trials, Case–Control and Cohort Studies. The findings of this Systematic Review and Meta‐Analysis led the authors to come to a common consensus that though dietary supplementation positively impacts certain cognition parameters, evidence was insufficient to bring statistical significance. This can be attributed to the small sample size of fourteen publications assessed in this study. Additionally, considerable differences in heterogeneity and the inability to perform sensitivity analysis on parameters because of limited sample size highlight the need for further research. Future directions should be aimed towards conducting additional long‐term and large‐scale studies, examining dose–response relationship to set an optimal dosage, considering factors such as genetic predispositions to identify specific populations that might benefit from Omega‐3 supplementation and explore potential synergistic effects of combining supplementation with other interventions, such as cognitive training, physical exercise, or other nutritional supplements, to enhance cognitive benefits.

## 8 CONCLUSION

Our Systematic review and meta‐analysis span from 2003 to 2023, covered a sample size of 14 studies, and 2766 participants explored the effects of omega‐3 fatty acid (particularly DHA) and nutritional supplement Souvenaid® on cognition, adverse events, and ventricular size in Alzheimer's disease. Our meta‐analysis did not find statistically significant differences between intervention and control groups for cognitive outcomes like ADCS‐ADL, ADAS‐cog, and MMSE scores. Ventricular volume analysis showed a nonsignificant trend in reduced decline with the Souvenaid® intervention group. The CDR scale analysis suggested that nutritional intervention may slow cognitive decline. Adverse effects from the omega‐3 supplementation were minimal and comparable to those of the control groups. While these findings contribute to the existing body of evidence on omega‐3 and Souvenaid® in AD, the provide inconclusive evidence of cognitive improvement. However, results suggest a potential beneficial effect in slowing cognitive decline and emphasize the need for further research to develop a personalized treatment proposal for individual patient presentation. Strengths of our review include robust methodologies and comprehensive analysis of multiple studies. It is imperative to acknowledge the limitations of this, such as the limited sample size, study heterogeneity, and publication bias. Future research should focus on several factors, including a larger, diverse sample size with an extended follow‐up period, elucidating the underlying mechanism, duration, dose of omega‐3 supplementation, and observation of Potential interaction between interventions. Although this review does not conclusively establish the efficacy of omega‐3 and Souvenaid® in enhancing cognition in AD patients, it sets the context for further investigation of a more personalized treatment approach. In doing so, it opens avenues for potential future benefits in managing cognitive decline associated with a debilitating condition called Alzheimer's disease.

## FUNDING INFORMATION

The study was unfunded, and there are no competing financial disclosures.

## CONFLICT OF INTEREST STATEMENT

The authors declare no conflict of interest.

## ETHICS STATEMENT

Approval of the research protocol by an Institutional Reviewer Board: N/A.

Informed Consent: N/A.

Registry and the Registration No. of the study/trial: N/A.

Animal Studies: N/A.

## PROTOCOL REGISTRATION

The protocol for this review has been registered on PROSPERO databases under the following available ID: CRD42024507453.

## Supporting information


Data S1.

## Data Availability

All descriptive variables are openly available in the articles of the studies that are cited in this manuscript.

## References

[1] Zhang XX , Tian Y , Wang ZT , Ma YH , Tan L , Yu JT . The epidemiology of Alzheimer's disease modifiable risk factors and prevention. J Prev Alzheimers Dis. 2021;8:313–321.34101789 10.14283/jpad.2021.15

[2] Knopman DS , Amieva H , Petersen RC , Chételat G , Holtzman DM , Hyman BT , et al. Alzheimer disease. Nat Rev Dis Primers. 2021;7:33. 10.1038/S41572-021-00269-Y 33986301 PMC8574196

[3] Silva MVF , Loures CDMG , Alves LCV , De Souza LC , Borges KBG , Carvalho MDG . Alzheimer's disease: risk factors and potentially protective measures. J Biomed Sci. 2019;26:33. 10.1186/S12929-019-0524-Y 31072403 PMC6507104

[4] 2023 Alzheimer's disease facts and figures. Alzheimers Dement. 2023;19:1598–1695. 36918389 10.1002/alz.13016

[5] Welty FK . Omega‐3 fatty acids and cognitive function. Curr Opin Lipidol. 2023;34:12–21. 36637075 10.1097/MOL.0000000000000862 PMC11878108

[6] Bellenguez CE , Küçükali F , Jansen IE , Kleineidam L , Moreno‐Grau S , Amin N , et al. New insights into the genetic etiology of Alzheimer's disease and related dementias. Nat Genet. 2022;54:412–436. 35379992 10.1038/s41588-022-01024-z PMC9005347

[7] Rajan KB , Weuve J , Barnes LL , McAninch EA , Wilson RS , Evans DA . Population estimate of people with clinical Alzheimer's disease and mild cognitive impairment in the United States (2020‐2060). Alzheimers Dement. 2021;17:1966–1975. 34043283 10.1002/alz.12362 PMC9013315

[8] Van Wijk N , Broersen LM , De Wilde MC , Hageman RJJ , Groenendijk M , Sijben JWC , et al. Targeting synaptic dysfunction in Alzheimer's disease by administering a specific nutrient combination. J Alzheimers Dis. 2014;38:459–479. 23985420 10.3233/JAD-130998

[9] Surette ME . The science behind dietary omega‐3 fatty acids. CMAJ. 2008;178:177–180. 18195292 10.1503/cmaj.071356 PMC2174995

[10] Hashimoto M , Hossain S . Neuroprotective and ameliorative actions of polyunsaturated fatty acids against neuronal diseases: beneficial effect of docosahexaenoic acid on cognitive decline in Alzheimer's disease. J Pharmacol Sci. 2011;116:150–162. 21606627 10.1254/jphs.10r33fm

[11] Wada M , DeLong CJ , Hong YH , Rieke CJ , Song I , Sidhu RS , et al. Enzymes and receptors of prostaglandin pathways with arachidonic acid‐derived versus eicosapentaenoic acid‐derived substrates and products. J Biol Chem. 2007;282:22254–22266. 17519235 10.1074/jbc.M703169200

[12] Hashimoto M , Maekawa M , Katakura M , Hamazaki K , Matsuoka Y . Possibility of polyunsaturated fatty acids for the prevention and treatment of neuropsychiatric illnesses. J Pharmacol Sci. 2014;124:294–300. 24561447 10.1254/jphs.13r14cp

[13] Vedin I , Cederholm T , Freund Levi Y , Basun H , Garlind A , Faxén Irving G , et al. Effects of docosahexaenoic acid–rich n−3 fatty acid supplementation on cytokine release from blood mononuclear leukocytes: the OmegAD study. Am J Clin Nutr. 2008;87:1616–1622. 18541548 10.1093/ajcn/87.6.1616

[14] Melo van Lent D , Egert S , Wolfsgruber S , Kleineidam L , Weinhold L , Wagner‐Thelen H , et al. Eicosapentaenoic acid is associated with decreased incidence of Alzheimer's dementia in the oldest old. Nutrients. 2021;13:461. 33573174 10.3390/nu13020461 PMC7912244

[15] Page MJ , McKenzie JE , Bossuyt PM , Boutron I , Hoffmann TC , Mulrow CD , et al. The PRISMA 2020 statement: an updated guideline for reporting systematic reviews. BMJ. 2021;372:n71. 10.1136/BMJ.N71 33782057 PMC8005924

[16] Calderon Martinez E , Flores Valdés JR , Castillo JL , Castillo JV , Blanco Montecino RM , Morin Jimenez JE , et al. 10 steps to conduct a systematic review. Cureus. 2023;15:e51422. 10.7759/cureus.51422 38299136 PMC10828625

[17] Ouzzani M , Hammady H , Fedorowicz Z , Elmagarmid A . Rayyan‐a web and mobile app for systematic reviews. Syst Rev. 2016;5:210. 10.1186/S13643-016-0384-4 27919275 PMC5139140

[18] Lo CKL , Mertz D , Loeb M . Newcastle‐Ottawa scale: comparing reviewers' to authors' assessments. BMC Med Res Methodol. 2014;14:45. 24690082 10.1186/1471-2288-14-45 PMC4021422

[19] Cochrane Handbook for Systematic Reviews of Interventions | Cochrane Training. Accessed October 25, 2023. https://training.cochrane.org/handbook/current

[20] Wells G , Shea B , O'Connell D , Peterson J , Welch V . The Newcastle‐Ottawa scale (NOS) for assessing the quality of nonrandomised studies in meta‐analyses. 2000. Accessed February 13, 2024. https://scholar.archive.org/work/zuw33wskgzf4bceqgi7opslsre/access/wayback/http://www3.med.unipmn.it/dispense_ebm/2009‐2010/Corso%20Perfezionamento%20EBM_Faggiano/NOS_oxford.pdf

[21] R: The R Project for Statistical Computing. Accessed February 14, 2024. https://www.r‐project.org/

[22] DerSimonian R , Laird N . Meta‐analysis in clinical trials. Control Clin Trials. 1986;7:177–188. 3802833 10.1016/0197-2456(86)90046-2

[23] Higgins JPT , Altman DG , Gøtzsche PC , Jüni P , Moher D , Oxman AD , et al. The Cochrane Collaboration's tool for assessing risk of bias in randomised trials. BMJ. 2011;343:d5928. 10.1136/BMJ.D5928 22008217 PMC3196245

[24] Egger M , Smith GD , Schneider M , Minder C . Bias in meta‐analysis detected by a simple, graphical test. BMJ. 1997;315:629–634. 9310563 10.1136/bmj.315.7109.629 PMC2127453

[25] Begg CB , Mazumdar M . Operating characteristics of a rank correlation test for publication bias. Biometrics. 1994;50:1088–1101. 7786990

[26] McGuinness LA , Higgins JPT . Risk‐of‐bias VISualization (robvis): an R package and shiny web app for visualizing risk‐of‐bias assessments. Res Synth Methods. 2021;12:55–61. 32336025 10.1002/jrsm.1411

[27] Freund‐Levi Y , Eriksdotter‐Jönhagen M , Cederholm T , Basun H , Faxén‐Irving G , Garlind A , et al. Omega‐3 fatty acid treatment in 174 patients with mild to moderate Alzheimer disease: OmegAD study: a randomized double‐blind trial. Arch Neurol. 2006;63:1402–1408. 17030655 10.1001/archneur.63.10.1402

[28] Soininen H , Solomon A , Visser PJ , Hendrix SB , Blennow K , Kivipelto M , et al. 24‐month intervention with a specific multinutrient in people with prodromal Alzheimer's disease (LipiDiDiet): a randomised, double‐blind, controlled trial. Lancet Neurol. 2017;16:965–975. 29097166 10.1016/S1474-4422(17)30332-0 PMC5697936

[29] Soininen H , Solomon A , Visser PJ , Hendrix SB , Blennow K , Kivipelto M , et al. 36‐month LipiDiDiet multinutrient clinical trial in prodromal Alzheimer's disease. Alzheimers Dement. 2021;17:29–40. 32920957 10.1002/alz.12172 PMC7821311

[30] Kamphuis PJGH , Verhey FRJ , Olde Rikkert MGM , Twisk JWR , Swinkels SHN , Scheltens P . Efficacy of a medical food on cognition in Alzheimer's disease: results from secondary analyses of a randomized, controlled trial. J Nutr Health Aging. 2011;15:720–724.21968871 10.1007/s12603-011-0105-6

[31] Yamazaki T , Nagata K , Takano D , Saito M , Shinoda T , Muraoka R , et al. Effect of polyunsaturated fatty acids on the evolution of cognitive ability in elderly patients with Alzheimer's disease. J Cardiovasc Disord. 2015;2:1007.

[32] Boston PF , Bennett A , Horrobin DF , Bennett CN . Ethyl‐EPA in Alzheimer's disease—a pilot study. Prostaglandins Leukot Essent Fatty Acids. 2004;71:341–346. 15380822 10.1016/j.plefa.2004.07.001

[33] Kamphuis PJGH , Verhey FRJ , Olde Rikkert MGM , Twisk JWR , Swinkels SHN , Scheltens P . Effect of a medical food on body mass index and activities of daily living in patients with Alzheimer's disease: secondary analyses from a randomized, controlled trial. J Nutr Health Aging. 2011;15:672–676.21968863 10.1007/s12603-011-0339-3

[34] Scheltens P , Kamphuis PJGH , Verhey FRJ , Olde Rikkert MGM , Wurtman RJ , Wilkinson D , et al. Efficacy of a medical food in mild Alzheimer's disease: a randomized, controlled trial. Alzheimers Dement. 2010;6:1–10.e1. 10.1016/J.JALZ.2009.10.003 20129316

[35] Eriksdotter M , Vedin I , Falahati F , Freund‐Levi Y , Hjorth E , Faxen‐Irving G , et al. Plasma fatty acid profiles in relation to cognition and gender in Alzheimer's disease patients during oral omega‐3 fatty acid supplementation: the OmegAD study. J Alzheimers Dis. 2015;48:805–812. 26402079 10.3233/JAD-150102

[36] Shah RC , Kamphuis PJ , Leurgans S , Swinkels SH , Sadowsky CH , Bongers A , et al. The S‐connect study: results from a randomized, controlled trial of Souvenaid in mild‐to‐moderate Alzheimer's disease. Alzheimers Res Ther. 2013;5:59. 24280255 10.1186/alzrt224 PMC3978853

[37] Quinn JF , Raman R , Thomas RG , Yurko‐Mauro K , Nelson EB , Van Dyck C , et al. Docosahexaenoic acid supplementation and cognitive decline in Alzheimer disease: a randomized trial. JAMA. 2010;304:1903–1911. 21045096 10.1001/jama.2010.1510 PMC3259852

[38] Freund‐Levi Y , Basun H , Cederholm T , Faxén‐Irving G , Garlind A , Grut M , et al. Omega‐3 supplementation in mild to moderate Alzheimer's disease: effects on neuropsychiatric symptoms. Int J Geriatr Psychiatry. 2008;23:161–169. 17582225 10.1002/gps.1857

[39] Chiu CC , Su KP , Cheng TC , Liu HC , Chang CJ , Dewey ME , et al. The effects of omega‐3 fatty acids monotherapy in Alzheimer's disease and mild cognitive impairment: a preliminary randomized double‐blind placebo‐controlled study. Prog Neuro‐Psychopharmacol Biol Psychiatry. 2008;32:1538–1544. 10.1016/j.pnpbp.2008.05.015 18573585

[40] Shinto L , Quinn J , Montine T , Dodge HH , Woodward W , Baldauf‐Wagner S , et al. A randomized placebo‐controlled pilot trial of omega‐3 fatty acids and alpha lipoic acid in Alzheimer's disease. J Alzheimers Dis. 2014;38:111–120. 24077434 10.3233/JAD-130722 PMC3886557

[41] Hooijmans CR , Pasker‐De Jong PCM , De Vries RBM , Ritskes‐Hoitinga M . The effects of long‐term omega‐3 fatty acid supplementation on cognition and Alzheimer's pathology in animal models of Alzheimer's disease: a systematic review and meta‐analysis. J Alzheimers Dis. 2012;28:191–209. 22002791 10.3233/JAD-2011-111217

[42] Wu S , Ding Y , Wu F , Li R , Hou J , Mao P . Omega‐3 fatty acids intake and risks of dementia and Alzheimer's disease: a meta‐analysis. Neurosci Biobehav Rev. 2015;48:1–9. 25446949 10.1016/j.neubiorev.2014.11.008

[43] Araya‐Quintanilla F , Gutiérrez‐Espinoza H , Sánchez‐Montoya U , Muñoz‐Yañez MJ , Baeza‐Vergara A , Petersen‐Yanjarí M , et al. Effectiveness of omega‐3 fatty acid supplementation in patients with Alzheimer disease: a systematic review and meta‐analysis. Neurologia. 2020;35:105–114. 28986068 10.1016/j.nrl.2017.07.009

